# The emerging role of miR‐19 in glioma

**DOI:** 10.1111/jcmm.13788

**Published:** 2018-08-02

**Authors:** Weihan Wang, Anling Zhang, Yubing Hao, Guangxiu Wang, Zhifan Jia

**Affiliations:** ^1^ Department of Neurosurgery Tianjin Medical University General Hospital Tianjin Neurological Institute Laboratory of Neuro‐Oncology Key Laboratory of Post‐Trauma Neuro‐Repair and Regeneration in Central Nervous System Ministry of Education Tianjin Key Laboratory of Injuries, Variations and Regeneration of Nervous System Tianjin China

**Keywords:** chemotherapy, glioma, miR‐19, radiotherapy, target gene

## Abstract

Glioma has been regarded as the most common, highly proliferative and invasive brain tumour. Advances in research of miRNAs in glioma are toward further understanding of the pathogenesis of glioma. MiR‐19, a member of miR‐17~92 cluster, was reported to play an oncogenic role in tumourigenesis. Here we review the identified data about the effect of miR‐19 on proliferation, apoptosis, migration and invasion of glioma cells, the target genes regulated by miR‐19, and correlation of miR‐19 with the sensitivity of glioma cells to chemotherapy and radiotherapy. It is concluded that miR‐19 plays an important role in the pathogenesis of glioma and can be a potential target for gene therapy of glioma.

## 1 INTRODUCTION

MicroRNAs, a class of endogenous non‐coding RNA with 18‐25 nucleotides, repress the expression of corresponding genes by binding to the 3′‐UTR region of target genes at post‐transcriptional level.^1^, ^2^ MiRNAs have been identified to participate in a variety of cell biological processes such as proliferation, apoptosis, migration and invasion.^3^, ^4^ During the development of cancer, miRNAs are dysregulated and play oncogenic or tumour suppressive role by enhancing or suppressing proliferation, invasion of tumour cells.^5^, ^6^ Thus, miRNA deregulation is one of the key mechanisms in glioma pathogenesis. The relevant miRs can be used as new targets of glioma therapy and provide clues for diagnosis.

This review will discuss the role of miR‐19 in glioma cell proliferation, apoptosis and migration and its effect on chemotherapy and radiotherapy of glioma.

**Figure 1:**
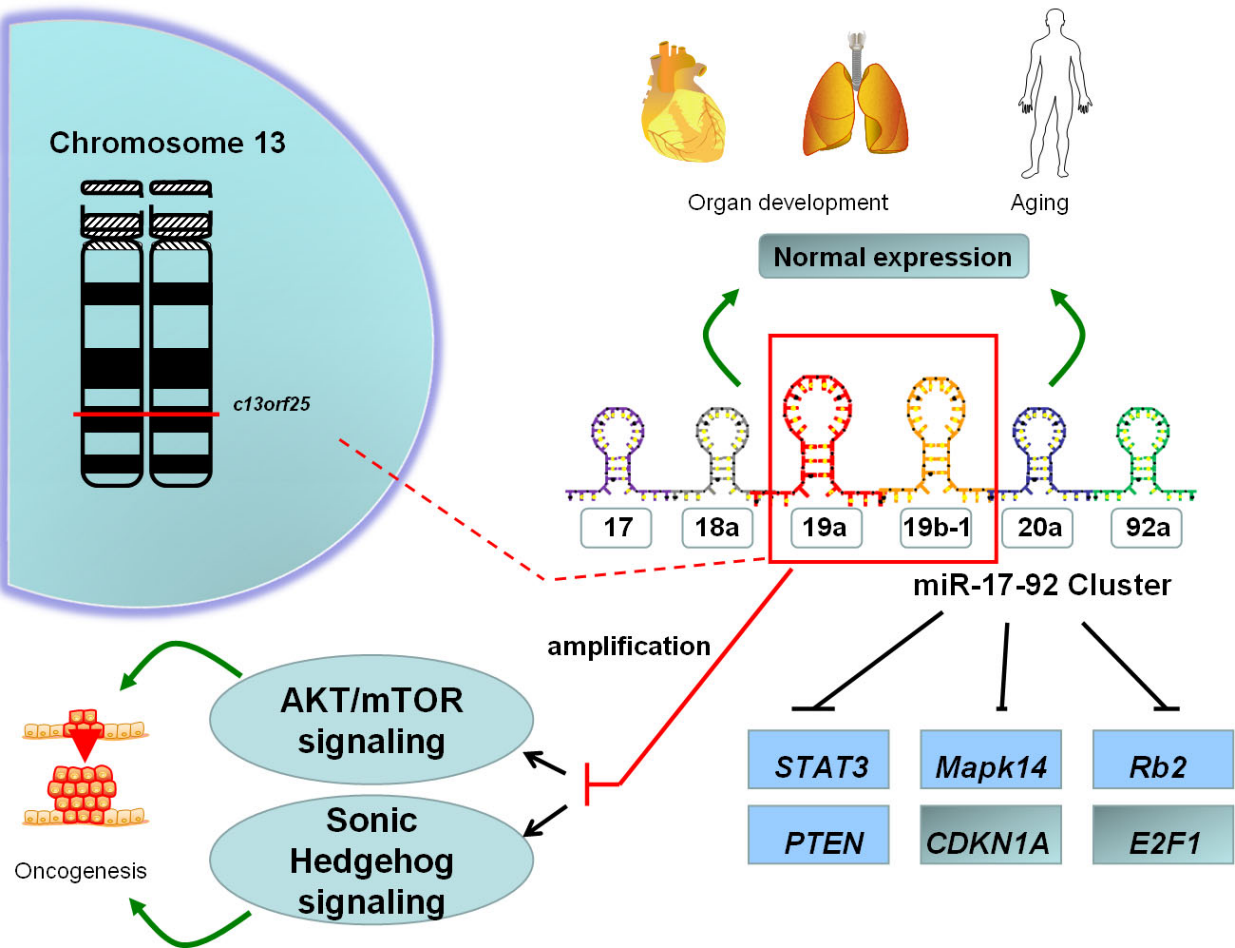
Sketch of miR‐17‐92 cluster and miR‐19

MiR‐19 is a member of miR‐17‐92 cluster, this cluster participates not only in the development of heart and lung,^7^, ^8^ but also in ageing and cancer.^9^ The target genes of miR‐17‐92 cluster have been experimentally identified so far including: STAT3, Mapk14^10^ and Rb2/p130.^11^ MiR‐17‐92 cluster plays an important role in tumourigenesis of thyroid cancer, leukaemia and lymphoma.^12^, ^13^, ^14^ The expression of miR‐17‐92 cluster is up‐regulated in glioma tissues. MiR‐17‐92 cluster inhibition decreases cell proliferation and induces apoptosis in glioblastoma spheroids culture by up‐regulating the expression of CDKN1A (cyclin‐dependent kinase inhibitor 1A), E2F1 and PTEN.^15^ MiR‐17‐92 cluster is regarded as the first miRNA cluster with oncogenic potential,^15^ the cluster includes 6 single mature miRNAs, miR‐19 has been supposed to be the key oncogenic miRNA among the six members of miR‐17‐92 cluster. MiR‐19 is located on chromosome 13 in *c13orf25* 16 and its expression is up‐regulated in bladder cancer, breast cancer, pancreatic cancer, gastric cancer and laryngeal squamous cell carcinoma.^17^, ^18^, ^19^, ^20^, ^21^ MiR‐19 promotes tumourigenesis by regulating target genes and related signalling pathways. In human B‐cell lymphomas, miR‐19 promotes tumour cell survival by inhibiting PTEN directly and activating AKT/mTOR pathway.^22^ Expression of miR‐19 is elevated in SHH medulloblastoma, a subgroup of medulloblastoma characeterized as constitutive activation of the Sonic Hedgehog pathway, anti‐miR‐19 treatment restrains proliferation of tumour cells and prolongs survival of tumour‐bearing mice^23^ (Figure 1).

## 2 MIR‐19 IN GLIOMA

The expression of miR‐19 is up‐regulated in glioma. In 75 archival paraffin‐embedded glioma specimens with different grades of malignancy and five normal control, miR‐19 is significantly up‐regulated in glioma tissues and positively correlates with the tumour grade.^24^ Increased expression level of miR‐19 is also detected in glioma cell lines.^25^ MiR‐19 is confirmed to participate in the process of glioma recurrence. A research report has demonstrated that miR‐19 is progression‐associated up‐regulation in patients who has been operated as WHO grade II originally and spontaneously progress to WHO grade IV secondary glioblastoma.^26^ This indicates that miR‐19 plays an important role in glioma progression. MiR‐19 is also regarded as prognostic biomarker of glioma, high expression of miR‐19 in patient's serum is associated with poor survival.^27^ MiR‐19 exerts its effect on biological characters of tumour cells through regulation on its target genes. It has been identified to regulate hundred of target genes in TargetScan and Pictar database, among them there are a few target genes have been experimentally confirmed such as PTEN,^24^ which play a significant role in glioma pathogenesis and progression. The effect of miR‐19 on glioma cell proliferation, apoptosis and migration and the impact of miR‐19 on chemotherapy and radiation therapy of glioma will be discussed separately as follows.

### 2.1 MiR‐19 and apoptosis

Studies demonstrate that miR‐19 inhibits apoptosis of glioma cells. Anti‐miR‐19 (antisense oligonucleotide of miR‐19) is introduced to knock down miR‐19 expression, apoptosis is induced in glioma cells.^28^ We have confirmed PTEN as the target gene of miR‐19 experimentally.^24^ PTEN plays a significant role as a tumour suppressor gene that induces glioma cell apoptosis and as a negative regulator of PI3K/AKT pathway, whereas PI3K/AKT inhibits apoptosis through repressing JNK and p38, or promoting FoxM1 expression in glioma.^29^, ^30^ Wang also reported that miR‐19 was suppressed by resveratrol in glioma and induced apoptosis, PTEN up‐regulation and PI3K/AKT repression.^31^ It has been reported that p53 is up‐regulated when miR‐19 is inhibited in glioma cells.^31^ p53 is a key proapoptosis gene. p53 induces p53‐dependent apoptosis through enhanced expression of transcription targets including STAG1, PUMA and PERP.^32^, ^33^, ^34^ MiR‐19 expression has been identified up‐regulated in glioma as oncomiR,^24^ apoptosis inducing proteins such as PTEN (directly suppressed by miR‐19) and p53 (negative regulated by miR‐19) are suppressed, so miR‐19 reduces apoptosis to promote tumour cell survival. It has been reported that miR‐19 also inhibit apoptosis in SH‐SY5Y human neuroblastoma cells, transfection of miR‐19 inhibitor leads to induction of apoptosis and increases expression of apoptosis‐related proteins including PTEN, p53, Bax and caspase‐3, decreases the expression of Bcl‐2.^35^

### 2.2 MiR‐19 and cell proliferation

Inhibition of miR‐19 by anti‐miR‐19 results in diminished proliferation of glioma cell in vitro.^28^ MiR‐19 depresses growth of glioma cells by negative regulation of PTEN, which can inhibit glioma cell proliferation by suppressing PI3k/AKT pathway.^36^ It also has been reported that miR‐19 promotes glioma progression by directly suppressing PPARα (the peroxisome proliferator‐activated receptor α, PPARα).^37^ PPAR belongs to nuclear receptor family which includes three subtypes, ie, PPARα, PPARγ and PPARδ. PPARα is well known for its role in regulating lipid and glucose metabolism, cell proliferation, tumourigenesis and inflammation. PPARα can inhibit glioma cell proliferation and induce cell cycle arrest by promoting nuclear translocation of FoxO1, then increases the expression of a FoxO1‐dependent cell cycle‐related protein, p27^kip^ in glioma cells.^38^ The antitumour role of PPARα also can be mediated by direct and indirect antiangiogenic effect on tumour cells.^39^ MiR‐19 directly down‐regulates the expression of PPARα by binding to 3′‐UTR region of PPARα mRNA in gliomas. Inhibitory effect of PPARα on glioma cell proliferation is blocked by targeting regulation of miR‐19, progression of tumour is enhanced.^37^ p53 has also been identified to be the target gene of miR‐19 in Hela cell.^40^ In glioma, expression of p53 is correlated negatively with that of miR‐19.^31^ p53 can impose cell cycle arrest and inhibit cell growth by decreasing the expression of Cyclin E1 and CDK2.^41^, ^42^ MiR‐19 promotes glioma cell proliferation that might be through inhibition on p53. MiR‐19 also has been reported to promote tumour cell proliferation in other tumours such as pancreatic cancer, castration‐resistant prostate cancer^43^, ^44^ and suppress tumour growth in lung cancer^45^ as well.

### 2.3 MiR‐19 and cell migration

It has been demonstrated that knocking down miR‐19 suppresses migration of glioma cell,^28^ whereas overexpression of miR‐19 promotes glioma cell migration and invasion. Long nocoding RNA: MEG3 suppresses glioma cell migration by playing a role as competing endogenous RNA (ceRNA) of miR‐19.^46^ As to the target genes of miR‐19 relevant to glioma cell invasion, it has been reported that RUNX3 suppresses glioma cell invasion by depressing the transcription activity of β‐catenin/TCF4 and expression of downstream factors of β‐catenin/TCF4 such as c‐MYC and AKT1,^47^ so miR‐19 possibly promotes glioma cell migration by direct negative regulation on RUNX3. RhoB, another target gene of miR‐19, is a member of Rho GTPase family. It participates in diverse cellular process including actin organization, differentiation to block cell migration activity. Introduction of exogenous RhoB suppresses glioma cell mobility and invasiveness by reducing activation of PKC iota and PKB/AKT.^48^ MiR‐19 is reported to promote glioma cell invasion and migration by directly suppressing RhoB by miR‐19.^25^ In addition, leucine‐rich repeats and immunoglobulin‐like domains 1 (LRIG1): as one of identified target genes of miR‐19, are confirmed to participate in the regulation on glioma invasion.^49^ Overexpression of LRIG1 suppresses U251 malignant glioma cell migration and invasion by reducing MMP2/9 level.^50^ Besides, LRIG1 inhibits phosphorylation of MAPK, EGFR and AKT signalling molecules and affects biological behaviours including migration and invasion by inactivation of EGFR/AKT signalling pathway.^51^, ^52^ Thus, the other alternative pathway that MiR‐19 promoting glioma migration might by directly inhibiting LRIG1 expression and indirect activation of EGFR/AKT signalling pathway. Since miR‐19 might negatively regulate EGFR via LRIG1, the impact of EGFR on invasion and angiogenesis of glioma cell may be connected with miR‐19. However, the connection between miR‐19 and EGFR should be studied further. In summary, miR‐19 promotes glioma cell migration mainly through the negative regulation on RUNX3, RhoB and LRIG1. Mir‐19 also exerts different effects on cell migration and invasion in diverse tumour cells. It has been demonstrated that miR‐19 enhances invasiveness of colorectal tumour cells by regulating target gene TG2 (Transglutaminase‐2)^53^ and promotes cell metastasis in hepatocellular carcinoma.^54^ However, miR‐19 exerts inhibitory effect on the ability of breast cancer cell migration and invasion,^55^ and it also inhibits migration and invasion of colon cancer cell by suppressing MMP9 and tissue factor (TF).^56^

## 3 MIR‐19 AND TREATMENT OF GLIOMA

### 3.1 MiR‐19 may be a novel target for gene therapy of glioma

Gliomas are the most challenging malignant brain tumours. Current standard treatment includes surgical resection, followed by radiotherapy and chemotherapy, co‐administration of temozolomide (TMZ). However, the prognosis of the patients with high‐grade glioma is still poor and the medium survival of GBM patients is only 15 months. Hence, researches on new therapeutic option are becoming hotspots in glioma study. Molecular target therapy against specific genes is an expectation for glioma treatment. Molecular target drugs like EGFR inhibitors and VEGFR antibodies have been applied in clinical practice. More and more studies on searching new target genes, including microRNAs, for molecular target therapy are carried out.

Overexpression of miR‐19 has been demonstrated to promote proliferation and invasion of glioma cells,^25^ knocking down miR‐19 by RNAi blocks tumour growth, induces cell apoptosis,^28^ all these results indicating that miR‐19 plays an important role in glioma progression. It suggests that miR‐19 can be identified as a potential target gene for glioma therapy. Since multiple proteins including RUNX3,^47^ RhoB,^25^ LRIG1^49^ and PTEN^24^ have been proved to be the target genes of miR‐19, when miR‐19 is knocked down by miR‐19 inhibitor, the expression of its target genes will be regulated accordingly, so it is predictable that multiple genes therapeutic effect can be achieved if novel anti‐oncomiR‐19 measures are adopted.

### 3.2 MiR‐19 and radiotherapy and chemotherapy sensitivity of glioma

In order to improve the prognosis of patients, malignant glioma patients are given radiotherapy and chemotherapy simultaneously after surgical resection. However, resistance to radiotherapy and chemotherapy turn out to be the important source of glioma recurrence.

MiR‐19 has been reported to exert effects on drug resistance in diverse tumour chemotherapy. In breast cancer, miR‐19 expression is up‐regulated in MDR (multidrug resistance) cell lines compared with corresponding parent cell line, knocking down miR‐19 restores sensitivity of MDR cells to chemotherapeutic agents by up‐regulating the expression of PTEN and decreasing the expression of MDR‐related transporters: BCRP (breast cancer resistance protein), MDR (multidrug resistance protein), MDR‐1 (P‐glycoprotein) and MRP‐1 (multidrug resistance‐associated protein 1).^57^ In gastric cancer, miR‐19 decreases the sensitivity of tumour cell to chemotherapy by targeting PTEN and promoting expression of AKT, pAKT and MDR‐1.^58^ Our literature reviews indicate that there is no report about the relationship of miR‐19 and sensitivity of glioma cells to chemotherapy up to now. While some studies provide clue for us to predict the effect of miR‐19 which might exert on reaction of glioma cell to chemotherapeutic agents. In multidrug resistance glioablastoma cell lines, MDR protein 1 (multidrug resistance protein 1) serves as drug resistance to decrease chemotherapy sensitivity of cells, MDR protein 1 up‐regulation is PI3K/AKT pathway activation dependent.^59^ PTEN—the negative regulator of PI3K/AKT pathway: also improves the sensitivity of tumour cell to drug in glioma cells.^60^ MiR‐19 might trigger the activation of PI3K/AKT pathway and up‐regulate the expression of drug resistance gene‐MDR protein 1 by directly inhibiting PTEN, so mir‐19 probably participates in promoting drug resistance of gloma cells by targeting PTEN.

Leung has shown that miR‐19 is significantly up‐regulated in breast cancer cell MDA‐MB‐361 cells after exposed to radiation, indicating that miR‐19 appears to be radiation‐associated miRNA in breast cancer.^61^ Knocking down miR‐19 in SiHa cervical cancer cells, the radiotherapy sensitivity of SiHa cells is increased and cell proliferation is inhibited.^62^ Above studies demonstrate that miR‐19 is associated with radiosensitivity of tumour cells. However, research on the relationship between miR‐19 and radiotherapy in glioma is limited. Chaudhry has reported that miR‐19 is up‐regulated in both irradiated glioma cell line M059J which is deficient in DNA‐PK (DNA‐dependent protein kinase) and glioma cell line M059K with normal DNA‐PK activity, indicating that miR‐19 paritcipates in the reaction of different types of glioma cell lines to ionizing radiation.^63^ Some of the target genes of miR‐19 are related to the radiosensitivity of glioma cells. As previously reported, PTEN overexpression leads to an increase in sensitivity to ionizing radiation in glioma cells,^60^ LRIG1 enhances the sensitivity of radiotherapy in glioma cells by suppressing EGFR/AKT pathway.^64^ However, RhoB induces radioresistance in glioma cells.^65^ It has been identified that CtIP is the target gene of miR‐19 and CtIP plays a role in the DNA end resection and homologous recombination in response to DNA damage.^66^ Then we can infer that miR‐19 aberrant expression down‐regulating CtIP suppresses the repair of the most hazardous type of lesion, DNA double‐strand breaks induced by ionizing radiation, and may be helpful to enhance the effect of radiotherapy. Certainly, this assumption will be contradictory to the anti‐miR‐19 therapy for its oncogenic potential in glioma and the relationship between miR‐19 and radiation response of glioma needs to be investigated further.

## 4 CONCLUSION AND PERSPECTIVE

**Figure 2:**
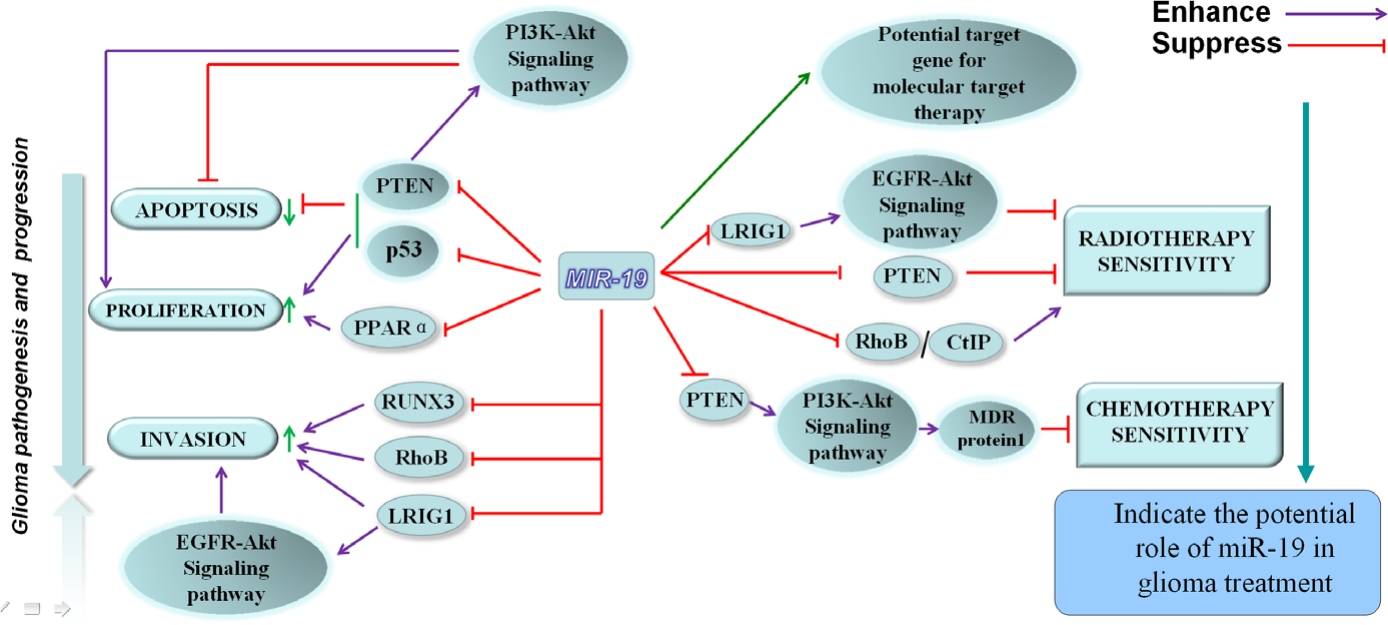
The role of miR‐19 in proliferation, migration, apoptosis and treatment of glioma

To date, studies on miR‐19 have been demonstrated to reduce apoptosis, promote proliferation and migration of glioma cells. Besides, it is associated with sensitivity of chemotherapy and radiotherapy of glioma cells. Intensive studies of the mechanism by which miR‐19 regulates proliferation and invasion of glioma cells will be helpful for providing a noval therapeutic target for glioma treatment. Standard care of glioma patients with combining surgery, chemotherapy and radiation therapy does not improve the survival rate of patients. Targeting oncomiRs seems to be the new trends in the development of miRNA therapeutic strategies. Thus, miR‐19 can be a new target in glioma treatment, or might regulate the effect of chemotherapy and radiotherapy by adjusting the sensitivity of glioma cells, which will provide a new clue for glioma therapy (Figure 2). Further study on miR‐19 will demonstrate its more roles in the pathogenesis of glioma, biomarker of prognosis and glioma treatment.

## CONFLICT OF INTEREST

The authors declare that we have no conflict of interest.
